# Chronic quercetin supplementation modulates cardiac function and signaling pathways in aged male Wistar rat hearts subjected to ischemia-reperfusion

**DOI:** 10.3389/fcvm.2026.1745113

**Published:** 2026-04-28

**Authors:** Jakub Strapec, Lucia Kindernay, Eva Kralova, Katarina Krsteva, Peter Pokreisz, Monika Bartekova, Tomas Rajtik, Kristina Ferenczyova

**Affiliations:** 1 Institute for Heart Research, Centre of Experimental Medicine, Slovak Academy of Sciences, Bratislava, Slovakia; 2 Department of Pharmacology and Toxicology, Faculty of Pharmacy, Comenius University, Bratislava, Slovakia; 3 Center for Biomedical Research and Translational Surgery, Medical University of Vienna, Vienna, Austria; 4 Institute of Physiology, Faculty of Medicine, Comenius University, Bratislava, Slovakia

**Keywords:** aging, apoptosis, autophagy, ischemia-reperfusion, quercetin

## Abstract

**Objectives:**

Quercetin (QCT), a natural polyphenol with antioxidant, anti-inflammatory, and antithrombotic properties, has shown cardioprotective effects in various *in vitro* and *in vivo* models of myocardial ischemia/reperfusion (I/R) injury. However, these effects have predominantly been demonstrated in young, healthy animals, which limit their translational potential, as patients with ischemic heart disease are typically middle-aged or older and frequently present with comorbidities. The present study aimed to evaluate the cardioprotective potential of chronic QCT treatment in aged rats.

**Methods:**

Male Wistar rats (20 months old at arrival) received QCT orally (20 mg/kg/day) for 6 weeks. After treatment, rats were euthanized and isolated hearts were perfused according to Langendorff, subjected to 30 min of global ischemia and 120 min of reperfusion. Cardiac function recovery was monitored during the first 40 min of reperfusion by assessment of electrical and mechanical parameters. Infarct size was determined by TTC staining at the end of reperfusion. In parallel groups, left ventricular tissue was collected immediately after treatment for Western blot (WB) analysis of regulatory proteins.

**Results:**

QCT administration improved electrical function, mainly by reducing QT and QTc intervals during reperfusion compared with controls. In contrast, QCT did not improve recovery of contractile function, and it did not reduce infarct size. WB analysis revealed a significantly increased Bcl-2/Bax ratio, suggesting attenuated apoptosis, while expression of other apoptotic and autophagy-related proteins remained unaffected.

**Conclusions:**

Compared with juvenile rat hearts, chronic QCT treatment exerts modest cardioprotective effects in aged hearts, characterized by improved electrical recovery and reduced pro-apoptotic signaling, independent of Reperfusion Injury Salvage Kinase (RISK) pathway or autophagy activation.

## 1 Introduction

Aging is a major determinant of cardiovascular health driving progressive structural and functional changes in the heart and vasculature. In addition to aging, cardiovascular disease (CVD) arises from a complex interplay of metabolic, lifestyle, and environmental factors that progressively contribute to cardiovascular risk across the lifespan (Ref. A). Metabolic disorders, particularly obesity, insulin resistance, type 2 diabetes mellitus, and dyslipidemia, markedly accelerate cardiovascular aging by promoting chronic low-grade inflammation, oxidative stress, and endothelial dysfunction (1, 2). Accordingly, the high prevalence of heart failure in elderly individuals is best understood as the consequence of lifelong exposures and metabolic burden that accumulate with age (3). In addition to cardiovascular aging, ischemia–reperfusion injury represents a major contributing factor in the development of heart failure. Long-term metabolic imbalance and lifestyle-related influences are known to increase myocardial susceptibility to ischemic stress by impairing mitochondrial function and metabolic adaptability (4). Cardiac ischemia-reperfusion (I/R) injury is a serious cardiovascular pathology, where both ischemia and reperfusion phases contribute to the ultimate extent of cardiomyocyte injury. I/R injury is associated with many different dysfunctions, including endothelial dysfunction, activation of innate immunity and inflammation, propagation through key molecular mediators such as reactive oxygen species (ROS), intracellular calcium overload and many more. During myocardial I/R, a burst of mitochondrial reactive oxygen species (ROS) upon reperfusion induces oxidative stress, leading to mitochondrial dysfunction, disruption of cellular homeostasis, and subsequent cardiomyocyte injury (5). Recently, it was shown that cardiac aging is on cellular level closely linked to senescence and metabolic reprogramming, involving processes such as impaired autophagy, oxidative stress, epigenetic alterations, chronic inflammation, and changes in myocyte contractile function (6). Such age-associated cellular alterations are modulated by epigenetic mechanisms that integrate long-term lifestyle and metabolic influences, contributing to maladaptive cardiac remodeling (7).

Autophagy, apoptosis, and pyroptosis are central mechanisms of cellular surveillance and programmed cell death, all of which are altered by aging and I/R injury. Autophagy normally preserves cardiomyocyte homeostasis by recycling damaged organelles, but it declines with age resulting in increased arrhythmia incidence and reduced stress tolerance (8). Metabolic overload and nutrient-sensing pathway dysregulation, including insulin/IGF-1, AMPK, and mTOR signaling, further suppress autophagic capacity in the aging heart (9). Apoptosis removes dysfunctional cells in a controlled manner, yet excessive activation contributes to cardiomyocyte loss during I/R (10). Pyroptosis, an inflammatory form of programmed death, further amplifies reperfusion-induced injury through caspase-1 and gasdermin-D signaling (11). Emerging evidence indicate that cardiac aging is tightly linked to activation of inflammasome-mediated pyroptosis, with anti-aging factors such as Klotho have been reported to mitigate D-galactose-induced cardiac senescence by suppressing the reactive oxygen species (ROS)/NOD-like receptor family, pyrin domain–containing 3 (NLRP3)/pyroptosis pathway (12). These inflammasome-driven processes, however, do not operate in isolation but are embedded within broader cellular stress-response networks that determine cell fate under metabolic and ischemic stress. A key molecular integrator of ischemic stress responses and age-related cell fate decisions, including autophagy, apoptosis, and pyroptosis, is the tumor suppressor protein p53. Chronic metabolic stress and nutrient excess promote maladaptive p53 activation, thereby leading to a shift in downstream p53-dependent signaling toward apoptosis and inflammation rather than cytoprotective repair pathways (13). In aged myocardium, such dysregulation of p53 activity is associated with increased vulnerability to ischemic stress (14, 15).

Importantly, several interventions, such as caloric restriction, sustained physical activity, rapamycin, spermidine, and resveratrol, have been shown to improve healthy aging by modulating these important pathways (16–18). In this context, antioxidants like quercetin (QCT), with well-documented antioxidant, anti-inflammatory, and metabolic benefits, may offer particular promise in elderly individuals, especially with regard to limiting myocardial damage in ischemia-reperfusion injury (19, 20). QCT has demonstrated cardioprotective effects against I/R injury in both *in vitro* and *in vivo* preclinical models, including isolated perfused hearts (21, 22), cultured cardiomyocytes (23), and various animal models of acute myocardial infarction (24, 25). However, these findings primarily obtained in young, healthy animals, have limited translational relevance to patients typically characterized by advanced age and comorbidities. QCT confers cardioprotection not only via its antioxidant properties but also by modulating key signaling pathways, including activation of RISK (26), regulation of apoptosis (27), necroptosis (23), and autophagy (28), as well as effects on macrophage polarization and Nrf2/mTOR signaling (29). Given that these mechanisms are strongly influenced by aging, assessing their regulation in aged hearts under I/R conditions is essential for understanding the translational potential of QCT and related cardioprotective strategies. For clarity, previously reported cardioprotective effects of quercetin across different experimental models are summarized in Supplementary Table S1.

## 2 Materials and methods

### 2.1 Chemicals

**Figure 1:**
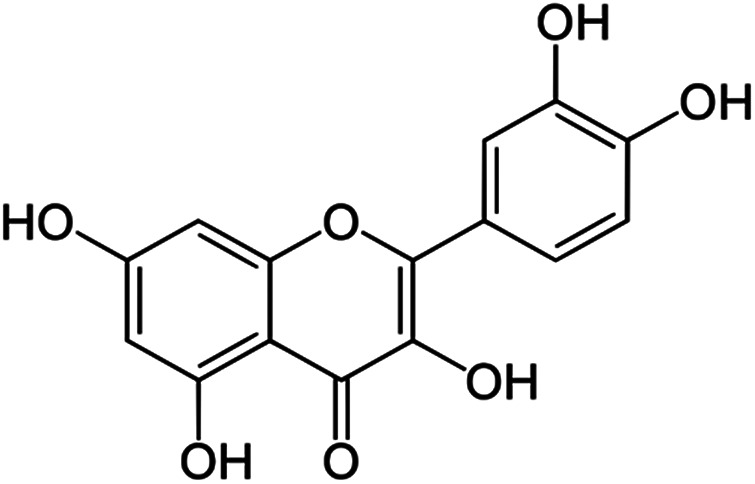
Structural formula of quercetin (3,5,7,3′,4′-pentahydroxyflavone) (30).

Quercetin (QCT, Figure 1) was obtained from Sigma Aldrich (catalog number Q4951, St. Louis, MO, USA).

### 2.2 Experimental model

Male Wistar rats, 20 months of age at the start of the experiment, were used to evaluate the potential cardioprotective effects of QCT. A total of 30 animals were housed under controlled environmental conditions (22 ± 2°C, relative humidity 45%–65%, 12:12 h light-dark cycle) with free access to standard rodent chow and water *ad libitum*. All animal procedures were performed in compliance with national regulations (State Veterinary and Food Administration of the Slovak Republic, legislation Nos. 377/2012 and 436/2012) governing the protection of animals used for scientific and educational purposes (Project No. 2237/18-221/3) and were approved by the institutional Ethics Committee of the Centre of Experimental Medicine, Slovak Academy of Sciences, Bratislava and in concordance with Health Guide for the Care and Use of Laboratory Animals (NIH Publication No. 85–23, revised 1996).

Animals were randomly assigned to two experimental groups: untreated controls (*n* = 15) and QCT-treated rats (*n* = 15). Within each group, 8 hearts were designated for *ex vivo* Langendorff perfusion experiments, whereas 7 were reserved for subsequent biochemical and molecular analyses. QCT was administered chronically for 6 weeks at a daily dose of 20 mg/kg. The compound was dissolved in ethanol (20 mg/mL) and delivered orally on a small piece of biscuit to ensure full ingestion as described previously (31). Throughout the treatment period, biometric parameters, including body weight (recorded weekly) and systolic blood pressure (measured before QCT initiation and after 6 weeks), were monitored.

**Figure 2:**
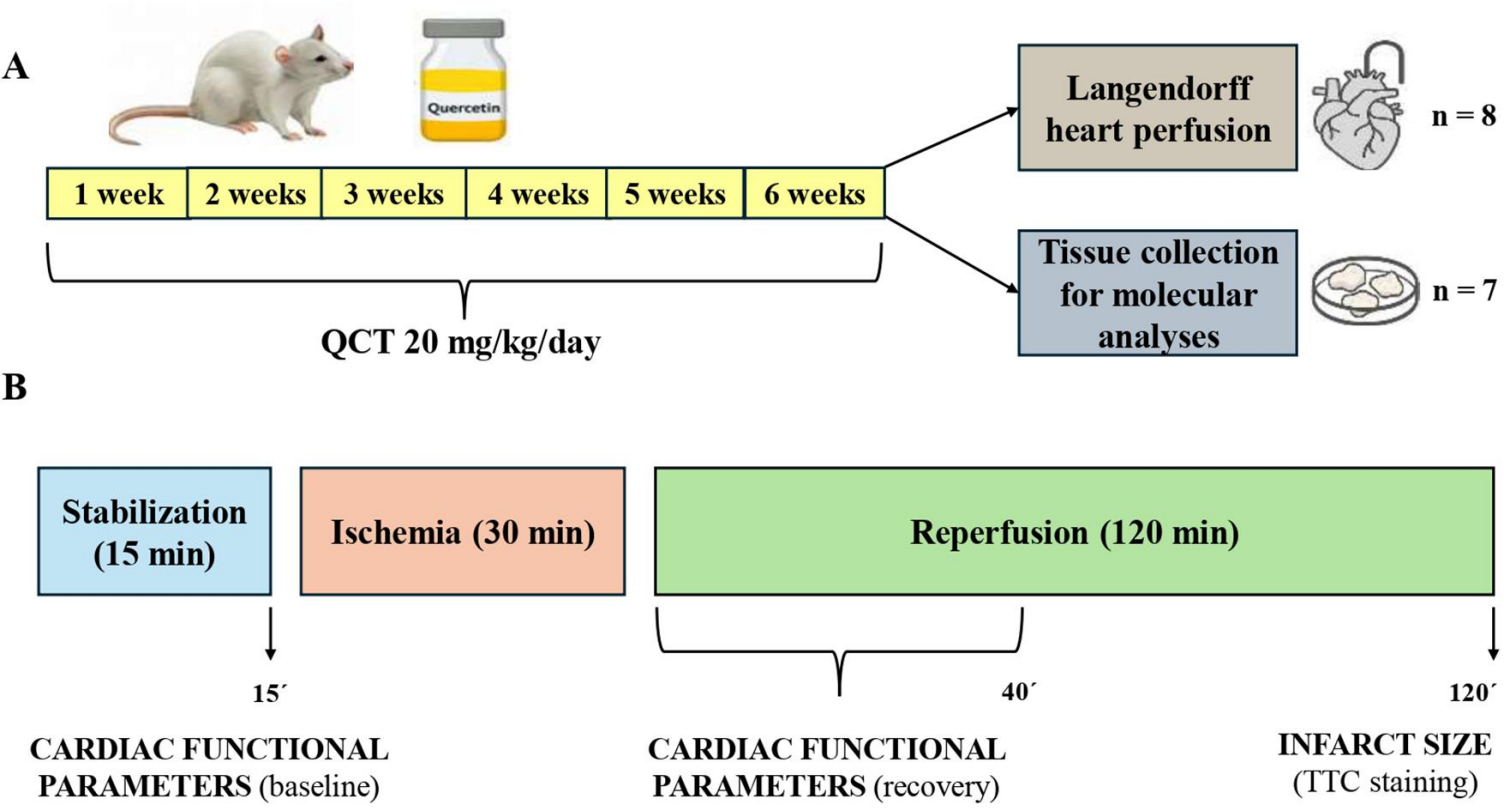
Overall experimental design **(A)** and ischemia/reperfusion protocol in isolated hearts **(B) (A)** overall experimental design. Twenty-month-old Wistar rats were randomly assigned to control (*n* = 15) and QCT-treated (*n* = 15) groups. Animals received daily QCT for 6 weeks. Hearts were then excised and either perfused at the Langendorff setup or processed for tissue sample collection. **(B)** Experimental protocol. Following stabilization, hearts were subjected to 30 min of global ischemia and 120 min of reperfusion. Functional and electrophysiological parameters were recorded throughout the protocol. QCT, quercetin; TTC, 2,3,5-triphenyltetrazolium chloride; n, number of animals.

At the conclusion of the treatment phase, animals were anesthetized with thiopental (50 mg/kg, i.p.; VUAB Pharma, Czech Republic) and heparinized (500 IU, i.p.; Zentiva, Slovakia) prior to heart excision. Hearts were rapidly (within 1 min) removed following thoracotomy and either immediately mounted for isolated heart perfusion using the Langendorff technique to assess ischemia/reperfusion (I/R) injury or snap-frozen in liquid nitrogen and stored at −80 °C for subsequent biochemical and molecular analyses (Figure 2).

### 2.3 Blood pressure measurement

The systolic blood pressure was measured by the non-invasive method of tail cuff plethysmography and the data were recorded by PowerLab 4/30 (ADInstruments, Budapest, Hungary) and evaluated as described previously (31). Animals were exposed to handling before measurements to avoid stress-induced BP elevation during the procedure. BP data were obtained from on average 3 measurements of the same animal at one time. The BP measurements were performed before the first QCT or vehicle application (beginning of week 1) and after the end of QCT/vehicle application (end of week 6).

### 2.4 Isolated heart perfusion at Langendorff setup for functional assessment

The Langendorff perfusion protocol was performed as previously described in our laboratory (21, 31, 32). Rats were anesthetized (thiopental 50 mg/kg, i.p) and heparinized (500 IU, i.p). Following confirmation of deep anesthesia (loss of pedal withdrawal reflex, typically within 2–4 min), hearts were excised within 60 s, immersed in ice-cold perfusion buffer, cannulated via the aorta, and mounted on a Langendorff apparatus (ADInstruments, Germany) for retrograde perfusion at a constant pressure of 73 mmHg and maintained at 37°C. The perfusion medium consisted of modified Krebs–Henseleit buffer (pH 7.4) continuously saturated with 95% O_2%_ and 5% CO_2_ throughout the experiment, containing (in mmol/L): NaCl 118.0, KCl 3.2, MgSO_4_ 1.2, NaHCO_3_ 25.0, KH_2_PO_4_ 1.18, CaCl_2_ 2.5, and glucose 7.0. The solution was filtered through a 5 μm porosity filter (Merck Millipore, Burlington, MA, USA) prior to perfusion.

Following mounting, hearts underwent a 15 min stabilization period under constant pressure and temperature to ensure stable hemodynamic parameters prior to ischemia. After stabilization, hearts were subjected to 30 min of normothermic global no-flow ischemia, achieved by complete cessation of perfusate flow, followed by 120 min of reperfusion. Functional recovery was assessed in the 40th min of reperfusion in those hearts whose recovered mechanical function persisted until 40th minute of reperfusion.

Bipolar electrocardiogram (ECG) was recorded via two stainless-steel electrodes attached to the ventricular apex and the aortic cannula. Left ventricular pressure was measured using a compliant, water-filled balloon inserted into the left ventricle through the left atrium, adjusted to achieve an end-diastolic pressure of 1–5 mmHg, and connected to a pressure transducer (MLP844, ADInstruments). Left ventricular developed pressure (LVDP; systolic minus diastolic pressure), maximal rates of pressure development and decay [±(dP/dt)_max_], heart rate (derived from ECG), and coronary flow were continuously monitored using a PowerLab/Chart system (ADInstruments). ECG parameter - QT interval duration (ms) was evaluated using the LabChart 7 ECG analysis module. Non-sinus beats were excluded, and 5 consecutive sinus beats per recording were analysed and averaged. Frequency-corrected QT intervals (QTc) were calculated using the modified Bazett's formula: QTcn-B = QT/(RR150)1/2 as described previously (33, 34).

### 2.5 Infarct size measurement

At the end of reperfusion, infarct size was assessed using 1% 2,3,5-triphenyltetrazolium chloride (TTC; Sigma-Aldrich, St. Louis, MO, USA) prepared in 0.1 M phosphate buffer (pH 7.4), following the established protocol (21). Hearts were incubated in TTC solution, fixed in 10% neutral buffered formaldehyde, sectioned transversely into 1 mm slices. The infarct size and the size of area at risk were measured by a computerized planimetric method. The infarct size was normalized to the size of area at risk, which in the model of global ischemia was the whole area of left ventricle. The analysis of IS was blinded.

### 2.6 Immunoblot analyses

Left ventricular tissue samples were homogenized and processed to obtain whole-cell protein lysates following a previously validated protocol (35). The lysates were subjected to SDS–PAGE, and separated proteins were subsequently transferred onto polyvinylidene difluoride (PVDF) membranes for immunoblot analysis.

For immunodetection, the following primary antibodies were used: anti-Akt1/2/3 (1:500), anti-PKC*ε* (1:500), anti-GSK-3*α*/*β* (1:500), anti-phospho-GSK-3β (Ser9) (1:500), and anti-GAPDH (1:4000) (Santa Cruz Biotechnology, Dallas, TX, USA); anti-phospho-Akt (Ser473) (1:1000), anti-phospho-eNOS (Ser1177) (1:1000), anti-BAX (1:200), anti-caspase-8 (1:1000), anti-LC3A/B (1:1000), anti-TNFR1 (1:1000), and anti-p53 (1:1000) (Cell Signaling Technology, Danvers, MA, USA); anti-Bcl-2 (1:200) (Invitrogen, Waltham, MA, USA); anti-Beclin-1 (1:1000) (Abcam, Cambridge, UK); and anti-eNOS (1:1000) (BD Biosciences, San Jose, CA, USA). Secondary antibodies were horseradish peroxidase (HRP)–conjugated anti-mouse and anti-rabbit IgG (1:50,000; Cell Signaling Technology, Danvers, MA, USA).

Membranes were incubated with the primary antibodies for 2 h at room temperature, followed by TBST washes (2 × 5–10 min). Then membranes were incubated with the secondary antibodies for −1 h at room temperature with gentle agitation (antibodies diluted in TBST or 1% PVP in TBST as appropriate), followed by TBST washes. Signals were detected using Clarity Western ECL Substrate (Bio-Rad Laboratories, USA) and visualized with an Amersham Imager 600 (Amersham, GE Healthcare, Chicago, IL, USA). Band intensities were quantified using ImageJ software (NIH, USA) or myECL Image Analysis software (Thermo Scientific, USA). For most immunoblots, total protein staining with Ponceau S (0.2% w/v in 3% w/v trichloroacetic acid) was used for normalization, whereas GAPDH immunodetection served as an additional loading control for selected targets. All reagents were obtained from Sigma-Aldrich (USA), Alfa-Aesar (USA), SERVA (Germany), CentralChem (Slovakia), Merck (USA), and Gentleham (UK).

### 2.7 Statistical analysis

All statistical analyses were performed using GraphPad Prism 8 (GraphPad Software, San Diego, CA, USA). Data are presented as mean ± standard deviation (SD). The assumption of normality was verified using the Shapiro–Wilk test prior to analysis. Comparisons between control and QCT-treated groups were conducted using an unpaired two-tailed Student's *t*-test. *P* ≤ 0.05 was considered statistically significant.

## 3 Results

### 3.1 Effect of QCT on biometric parameters and systolic blood pressure of aged rats

There were no differences in baseline biometric parameters including body weight and heart weight normalized to tibia length between control and QCT-treated groups before the treatment. No significant differences were detected in any of the measured biometric variables after 3 or 6 weeks of QCT treatment (Table 1).

**Table 1:** Biometric parameters of rats.

Variable	Before treatment		3 weeks of treatment		6 weeks of treatment		*P*-value
Group	Control	Quercetin	Control	Quercetin	Control	Quercetin	
BW (g)	553.6 ± 76.3	562.1 ± 42.6	564.0 ± 74.4	567.5 ± 40.4	579.1 ± 76.7	578.9 ± 44.6	ns
BW/TL (g/cm)	—	—	—	—	13.7 ± 1.8	13.8 ± 1.0	ns
HW (g)	—	—	—	—	2.0 ± 0.42	2.1 ± 0.1	ns
HW/TL (g/cm)	—	—	—	—	0.048 ± 0.010	0.049 ± 0.005	ns

Body weight was measured prior to, in the midterm, and after QCT treatment, heart weight and tibia length were measured after QCT treatment after scarifying the rats. BW, body weight; TL, tibia length; BW/TL, body weight normalized to tibia length; HW, heart weight; HW/TL, heart weight normalized to tibia length. Data are presented as mean ± SD. The number of animals was *n* = 15 per group. Statistical comparisons were performed between groups using Student's *t*-test.

**Figure 3:**
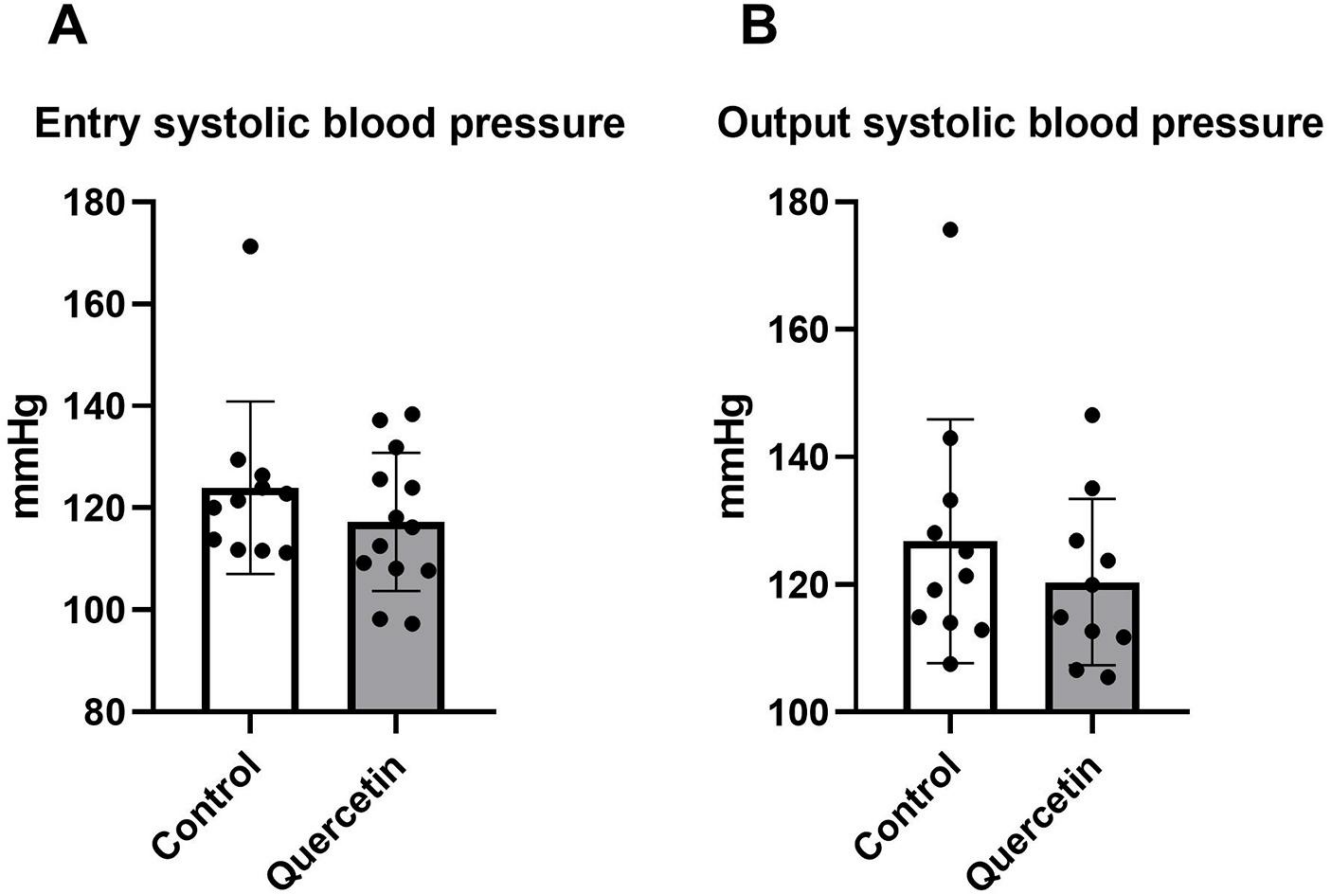
Effect of chronic QCT treatment on systolic blood pressure. Systolic blood pressure was assessed at two time points: **(A)** at entry, before the initiation of QCT treatment, and **(B)** at output, after 6 weeks of QCT administration. Each dot represents one animal. Data are presented as mean ± SD. Statistical significance was evaluated using unpaired Student's *t*-test.

As cardiac biometric indices remained unchanged, we subsequently evaluated systolic blood pressure. Systolic blood pressure was measured in aged Wistar rats at the beginning and at the end of the six-week experimental period. No significant differences were observed between the control and QCT-treated groups (*p* > 0.05, Figure 3).

### 3.2 Effect of QCT on electrical cardiac activity during post-ischemic reperfusion

**Figure 4:**
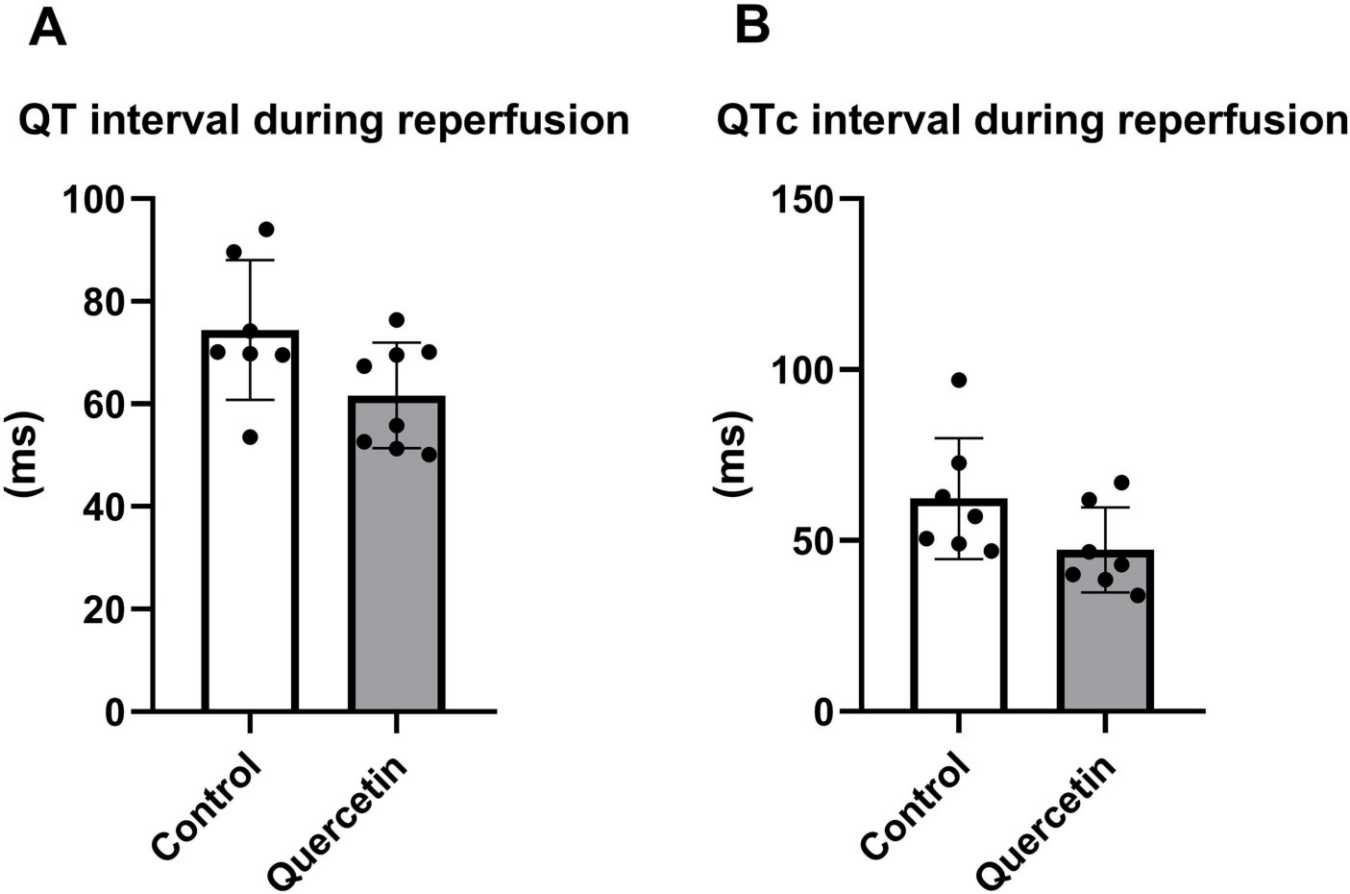
Effect of QCT treatment on QT and QTc intervals in aged Rat hearts. ECG recordings were analyzed in control and QCT-treated groups to assess **(A)** QT and **(B)** QTc intervals. Each dot represents one animal (*n* = 7–8 per group). Data are presented as mean ± SD. Statistical significance was evaluated using unpaired Student's *t*-test.

Ventricular repolarization parameters were evaluated from ECG recordings in isolated Langendorff-perfused hearts of aged Wistar rats. Our results demonstrated that chronic QCT administration improved cardiac electrical activity during post-ischemic reperfusion. This effect was manifested by a shortening of the QT interval which reached marginal significance (*P* = 0.0594) and a tendency toward QTc improvement (*P* = 0.0921) compared with the control group at comparable heart rate (Figure 4).

### 3.3 Effect of QCT on cardiac functional recovery and infarct size during reperfusion

Baseline cardiac functional parameters measured at the end of stabilization period prior to I/R did not differ significantly between control and QCT groups (Table 2).

**Table 2:** Pre-ischemic cardiac functional parameters.

Variable	Control	Quercetin	*P*-value
LVDP (mmHg)	88.5 ± 17.5	89 ± 7.9	ns
+(dP/dt)_max_ (mmHg/s)	1,713.4 ± 342.3	1,693.1 ± 135.1	ns
−(dP/dt)_max_ (mmHg/s)	1,200 ± 321.2	1,265 ± 128.1	ns
HR (beats/min)	190 ± 38.4	197.5 ± 30.9	ns
CF (ml/min)	11.3 ± 3.1	12.9 ± 2.3	ns

LVDP, left ventricular developed pressure; +(dP/dt)_max_, maximum rate of pressure development (mmHg/s); −(dP/dt)_max_, maximum rate of pressure decline (mmHg/s); HR, heart rate; CF, coronary flow. ns, not significant. Values are presented as mean ± SD (*n* = 8 per group). Statistical significance was evaluated using unpaired Student's *t*-test.

**Figure 5:**
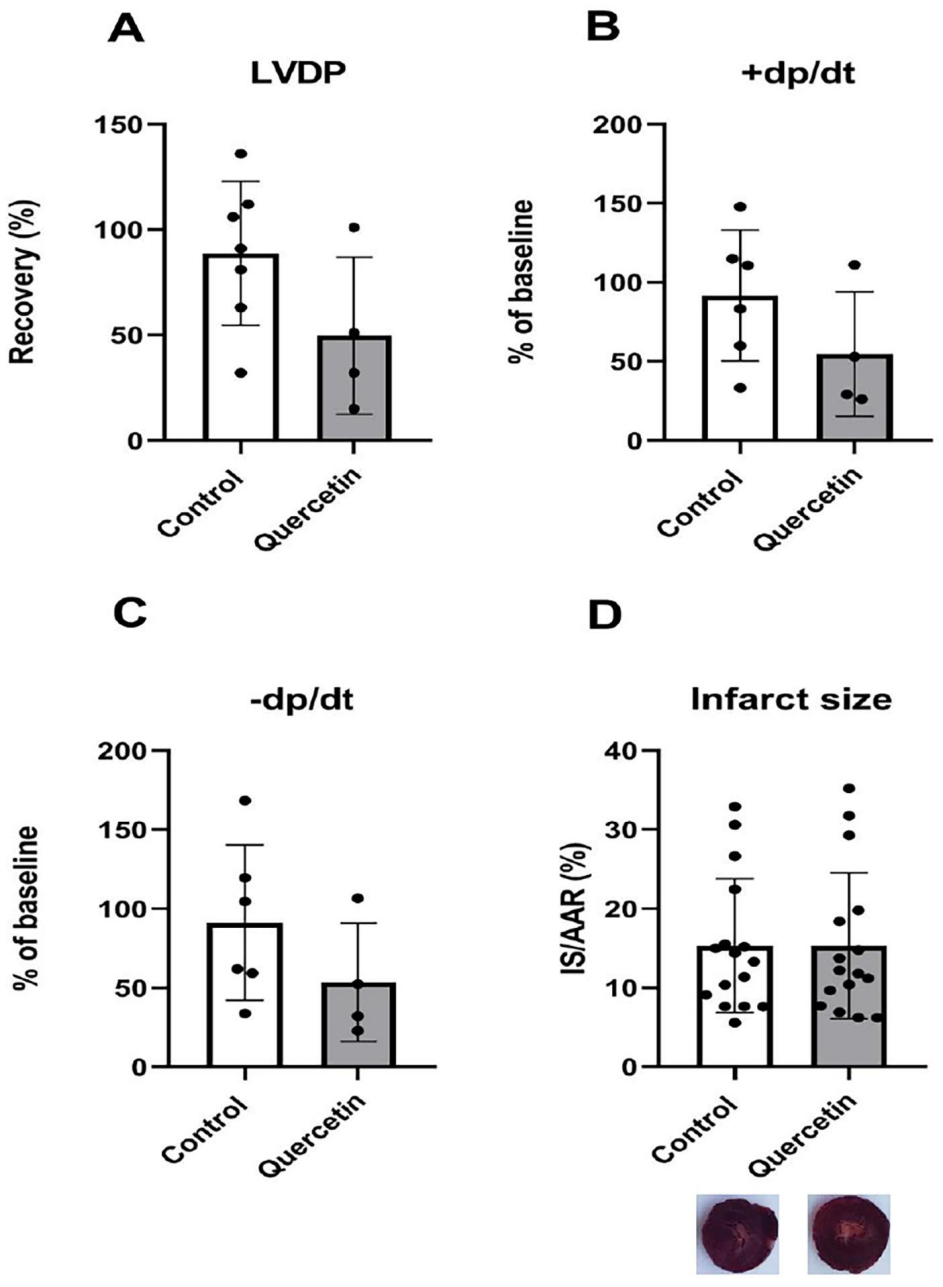
Effect of QCT treatment on post-ischemic functional recovery, +(dP/dt)_max_ and −(dP/dt)_max_ and infarct size in isolated langendorff-perfused rat hearts. **(A)** Left ventricular developed pressure (LVDP), **(B)** maximal rate of contraction +(dP/dt)_max_, and **(C)** maximal rate of relaxation −(dP/dt)_max_ measured at the 40th minute of reperfusion after 30 min of global ischemia, expressed as percentage of pre-ischemic baseline values. **(D)** Infarct size (IS) related to area at risk (AAR) determined by 2,3,5-triphenyltetrazolium chloride (TTC) staining after 2 h of reperfusion. In global ischemia, the entire left ventricle was considered the AAR. Representative TTC-stained heart sections from Control and Quercetin groups corresponding to infarct size analysis are shown. Each dot represents one animal. Data are presented as mean ± SD.

The effect of QCT treatment on post-ischemic recovery of cardiac function and infarct size was assessed in Langendorff-perfused hearts after 30 min global ischemia (Figure 5). Left ventricular developed pressure (LVDP), maximal rate of pressure development +(dP/dt)_max_, and maximal rate of pressure decay –(dP/dt)_max_ were measured in the 40th minute of reperfusion and expressed as percentages of pre-ischemic baseline values. Of 8 hearts in both groups that underwent the I/R procedure, 7 from the control group and 4 from the QCT treated group persisted their mechanical function until the 40th minute of reperfusion. Recovery of functional parameters did not differ significantly between QCT-treated and control groups. Infarct size was determined by 2,3,5-triphenyltetrazolium chloride (TTC) staining after 2 h of reperfusion and expressed as the ratio of infarct area to area at risk (IS/AAR).

### 3.4 Effect of QCT on RISK pathway proteins expression

**Figure 6:**
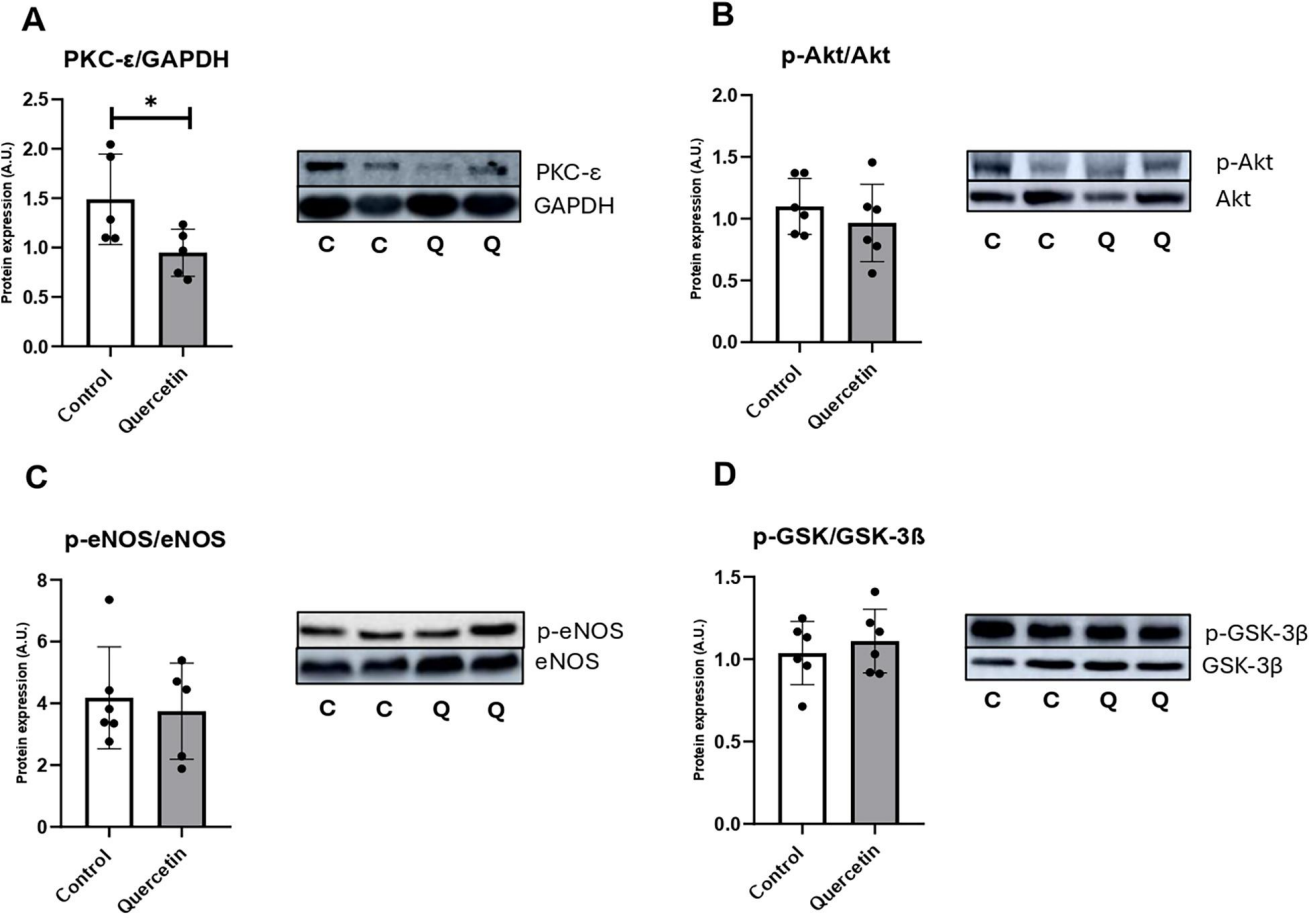
Expression of RISK pathway proteins in aged rat hearts. Western blot analysis of **(A)** total PKC-*ε* normalized to GAPDH, **(B)** phosphorylated Akt (Ser473)/total Akt, **(C)** phosphorylated eNOS (Ser1177)/total eNOS, and **(D)** phosphorylated GSK-3β (Ser9)/total GSK-3β in control and QCT-treated groups. Representative Western blot bands corresponding to each quantified protein are shown to the right of the graphs. Each dot represents one animal (*n* = 5-6/group). Data are presented as mean ± SD. Statistical significance was evaluated using unpaired Student's *t*-test. *p < 0.05.

The effect of QCT treatment on proteins involved in the Reperfusion Injury Salvage Kinases (RISK) pathway, namely Akt, eNOS, PKC-*ε*, and GSK-3β was evaluated. Statistical analysis revealed a significant reduction (*p* < 0.05) in PKC-*ε* expression normalized to GAPDH in the QCT-treated group compared with controls. For the other investigated targets (p-Akt/Akt, p-eNOS/eNOS, and p-GSK-3β/GSK-3β), no statistically significant differences were detected between groups suggesting that QCT did not change activation of the RISK pathway in 2-year-old rat hearts (Figure 6).

### 3.5 Effect of QCT on cell death–related pathways: apoptosis and autophagy

**Figure 7:**
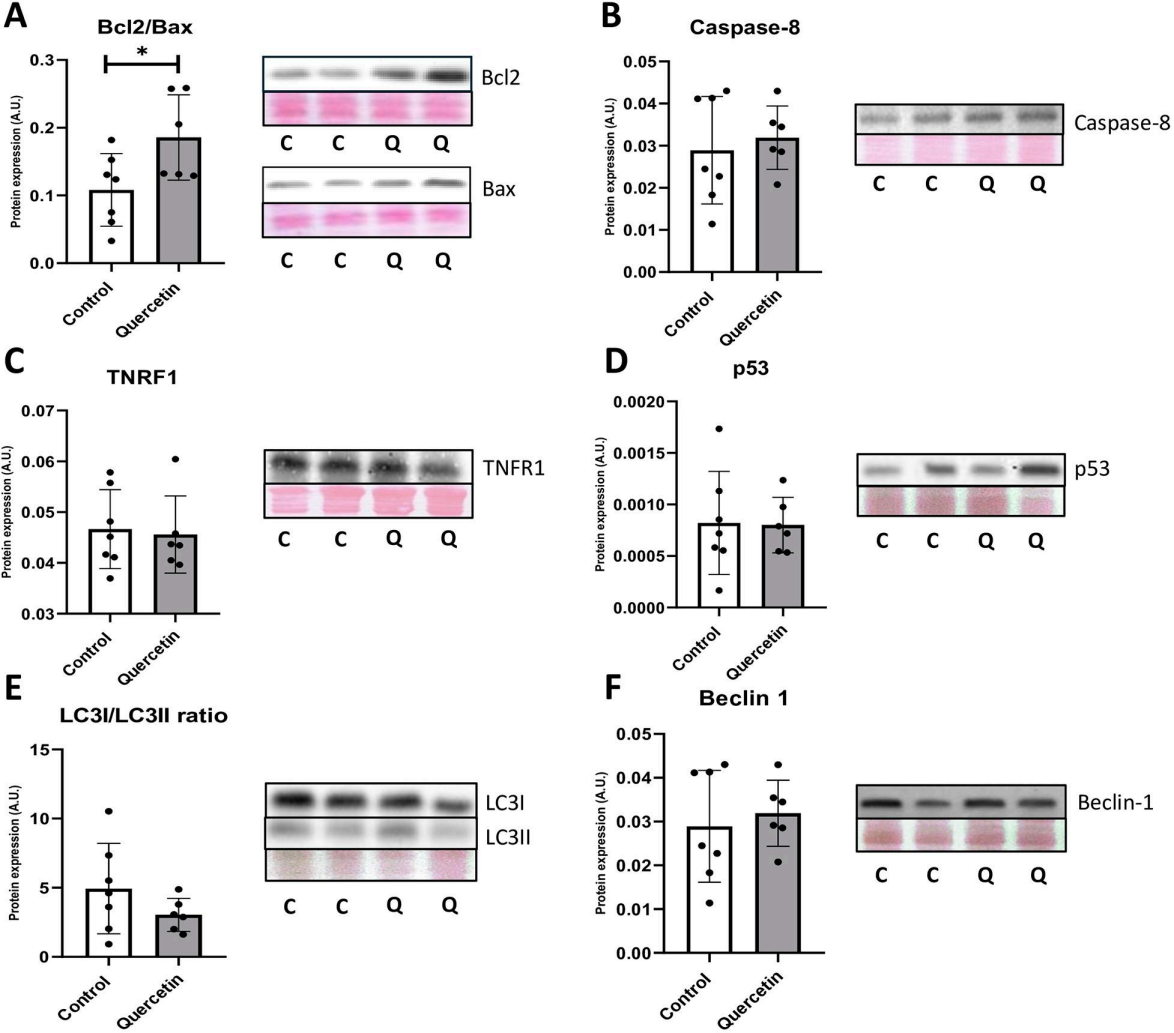
Western blotting analysis of apoptosis- and autophagy-related proteins in aged rat hearts following chronic QCT supplementation **(A)** Bcl-2/Bax ratio, **(B)** caspase-8, **(C)** TNFR1, **(D)** p53, **(E)** LC3-I/LC3-II ratio, and **(F)** beclin-1. Representative Western blot bands and corresponding Ponceau S total protein staining used for normalization are shown. Each dot represents one animal (*n* = 6–7 per group). Data are presented as mean ± SD. Statistical significance was determined using unpaired Student's *t*-test; *p < 0.05.

The effect of QCT treatment on apoptosis- and autophagy-related proteins was evaluated in rat hearts after QCT treatment (Figure 7). For apoptosis, analysis included TNFR1, Bcl-2/Bax ratio, and caspase-8. The Bcl-2/Bax ratio showed increase in the QCT-treated group compared with controls (*p* = 0.035), suggesting an anti-apoptotic shift in intrinsic apoptosis pathway. Expression of TNFR1 and caspase-8 remained unchanged suggesting no impact of QCT on extrinsic death receptor-mediated apoptosis pathway.

Regarding autophagy-related proteins, no statistically significant differences were detected between groups. The LC3-I/LC3-II ratio remained at similar levels in both groups; Beclin-1 levels were unchanged. In particular, p53, which is implicated in the regulation of both apoptosis and autophagy, also showed no significant differences between groups. All apoptosis- and autophagy-related proteins were quantified relative to total protein staining using Ponceau S.

## 4 Discussion

Aging is one of the main factors contributing to the development of cardiovascular diseases, including ischemic heart disease and myocardial infarction. Consequently, both acute cardiac events and chronic heart disease occur predominantly in older individuals. Paradoxically, however, most experimental animal studies aimed at identifying new cardioprotective strategies are conducted using young animals. In this study, we provided the first evidence of the cardiac effects of QCT in an aging animal model, using 2-year-old male Wistar rats. This age in rats corresponds to approximately 60 years in humans and is generally considered to be advanced age (36). Clinically, the average age of patients with acute myocardial infarction ranges from 60 to 70 years in men (male), which confirms the relevance of our model of an aging heart (37).

Our results demonstrated an improvement in the electrical function of isolated perfused heart of older rats during post-ischemic reperfusion as a result of chronic *in vivo* QCT treatment prior to I/R, which manifested itself in a shortening of the QT and QTc intervals in hearts treated with QCT. In contrast no significant alterations in post-ischemic contractile function were detected following QCT treatment. Chronic QCT treatment also showed a trend toward reduced apoptosis induction in left ventricular (LV) heart tissue, as evidenced by an increase in Bcl-2/Bax ratio; but without significant alterations in TNFR1 and caspase-8 expression, as well as no changes in autophagic proteins such as Beclin-1 and the LC3I/LC3II ratio. Further, QCT reduced PKC-*ε* expression in LV tissue, whereas expression of other proteins of main cardioprotective RISK pathway such as p-Akt/Akt, p-eNOS/eNOS and p-GSK-3β/GSK3-*β* remained unchanged. Overall, our findings suggest a modest cardioprotective effect of QCT in the aged heart, likely mediated at least partially due to the anti-apoptotic effect of QCT, but independent of its effects on autophagy and the RISK pathway. Furthermore, biometric parameters and systolic blood pressure did not differ between groups after QCT treatment, suggesting that the observed findings were not attributable to or associated with differences in body composition or blood pressure.

In our previous studies, we have demonstrated the potent cardioprotective effect of QCT on ischemia/reperfusion (I/R)-induced cardiac injury, both after acute administration *in vitro* and chronic administration *in vivo* in young animals (21, 32). Recently, other groups also confirmed the cardioprotective effects of QCT in young rats and mice (24, 25, 38). In addition, the cardioprotective effect of QCT has been observed in cultured cardiac cells exposed to simulated I/R injury *in vitro* (23). QCT has also been shown to protect against I/R injury in young animals co-treated with the anthracycline doxorubicin (DOX) (22) and in streptozotocin (STZ)-induced type 1 diabetic rats (39). However, the effect of QCT on cardiac I/R in the context of aging has not yet been investigated. Our study therefore provides novel insights into the cardioprotective potential of QCT in cardiac I/R, since acute myocardial infarction (AMI) and ischemic heart disease (IHD) occur predominantly in older individuals. However, the incidence in younger populations is increasing due to unhealthy lifestyles, chronic stress and adverse environmental factors. Our data indicate a slightly different and more modest cardioprotective effect of QCT compared with previous studies in young animals. While studies in young animals have mainly pointed to the strong cardioprotective effect of QCT and its derivatives on restoring cardiac mechanical function (21, 32), reducing infarct size (21), and preventing adverse post-infarction cardiac remodeling (24, 38) without significantly affecting the electrical function of the heart (32); the current study revealed significant improvements in electrical parameters of the heart (QT, QTc intervals) without significant changes in mechanical function or IS. These findings suggest that the effects of QCT on post-ischemic cardiac recovery may depend on age-related myocardial remodeling. In aged hearts, the observed improvement in QT/QTc recovery may reflect modulation of electrophysiological properties rather than direct effects on mechanical structures, while the precise molecular mechanisms remain to be clarified by future targeted studies.

The RISK pathway has been repeatedly identified as a key mediator of cardioprotection in I/R-induced cardiac injury, activated by various cardioprotective interventions both *in vivo* and *in vitro*, including its activation by ischemic conditioning strategies (40) and pharmacological cardioprotective treatments (41, 42), as well as by treatment with natural polyphenols such as catechins (43, 44), curcumin (45) or fisetin (46). Activation of the RISK pathway has also been reported to contribute to cardioprotective effects of QCT against I/R injury (26). In our study, we therefore examined the expression of proteins involved in the RISK pathway. Our results showed that the entire RISK pathway was not activated in left ventricular tissue by QCT treatment, as evidenced by unchanged expression/phosphorylation of the main proteins of this pathway, including Akt, eNOS, and GSK-3β. The only significant change observed was a decrease in PKC-*ε* expression attributable to QCT treatment. These findings are in consistency with our previous study, in which QCT applied to ZDF rats with T2DM neither did not activate the cardiac RISK pathway nor did not protect the heart from I/R damage (31). On the other hand, activation of Akt kinase and related signaling pathways has been found to be associated with the beneficial effects of QCT and its derivatives in I/R (26, 47, 48). We therefore hypothesize that the inability of QCT to protect post-ischemic cardiac function recovery and reduce MI in older animals may be at least partially attributable to insufficient activation of the RISK pathway in our study. However, the precise role of the RISK pathway in the cardiac effects of QCT requires further investigation in various animal models of cardiac I/R injury.

QCT has been consistently shown to inhibit apoptosis in various models of cardiac injury, including I/R injury (27, 38), anthracycline-induced cardiotoxicity (22) and diabetes-associated myocardial damage (49, 50), where this anti-apoptotic effect was associated with cardioprotection. In agreement with previous reports, we observed that QCT increased the Bcl-2/Bax ratio, shifting the balance toward an anti-apoptotic effect. However, upstream apoptotic mediators such as TNFR1 and caspase-8 remained unchanged, indicating only partial modulation of the apoptotic cell death. Previous studies have shown that reduced Bax/Bcl-2 correlates with improved I/R tolerance (47, 51), but in our model of ageing this shift did not improve mechanical function or infarct size. This suggests that in aged myocardium inhibition of apoptosis alone is insufficient to achieve cardioprotection or that the anti-apoptotic effect of QCT was limited under age-related stress conditions. Since QCT suppresses apoptosis in other cardiac injury models outside of I/R (22, 50), apoptosis inhibition may represent a general cytoprotective response rather than an I/R-specific mechanism.

However, apoptosis represents only one branch of a highly coordinated programmed cell death network. Increasing evidence indicates that cardiomyocyte fate, particularly in aging, is shaped by upstream regulators rather than isolated pathways. Among these, p53 functions as a central integrator of cellular stress responses influencing whether cells activate adaptive survival mechanisms or undergo cell death (14, 15). Under moderate stress, p53 may promote protective autophagy via AMPK activation and mTOR inhibition (17), enabling clearance of damaged organelles. Autophagy plays a crucial role in stress tolerance, yet its efficiency declines with age (8), contributing to increased vulnerability to I/R. On the contrary, sustained or excessive p53 activation in aged myocardium suppresses autophagy and enhances pro-apoptotic signaling, shifting the balance toward cell death (14).

In our study, QCT did not alter p53 expression or autophagy-related proteins (LC3-I/II, Beclin-1), indicating that upstream regulators of cell survival and death pathways were not detectably modulated at baseline following chronic treatment. Given that these molecular analyses were performed prior to I/R, they cannot establish whether modulation of these pathways is necessary or sufficient for functional cardioprotection. This might be of particular interest because many cardioprotective interventions in young animals depend on autophagy induction to preserve mitochondrial integrity and function (16, 28). The absence of autophagy activation may therefore explain why QCT failed to improve mechanical recovery or reduce infarct size in aged hearts, even though it increased Bcl-2/Bax ratio. Importantly, aging is associated with profound mitochondrial vulnerability and structural remodeling, including fibrosis, which may further limit the translation of favorable downstream apoptotic shifts into functional protection.

Interestingly, QCT improved electrical recovery (shortened QT/QTc intervals) without affecting mechanical performance. Previous studies have shown that decreased apoptosis can preferentially stabilize electrophysiological function (52, 53), whereas increased apoptosis contributes to QT prolongation (54, 55). Thus, even a mild reduction in pro-apoptotic signaling may be more significant on electrical than mechanical outcomes. In contrast, improvement of contractile dysfunction might require broader engagement of survival pathways such as autophagy (56) or RISK-mediated signaling (57), which were not activated in our study.

Taken together, these findings support a model in which programmed cell death in aging is an integrated process where impaired autophagy and enhanced apoptosis coexist. QCT attenuated intrinsic downstream apoptosis, which may explain the improvement in electrical stability, but failed to modulate p53-mediated signaling or restore autophagy, limiting its overall cardioprotective effect. Therefore, more effective strategies should aim to reestablish the p53–autophagy–apoptosis axis, potentially through combination therapies that enhance autophagy or directly modulate p53 activity, to achieve more effective cardioprotection in the aged heart.

## 5 Conclusion

Our findings indicate that chronic QCT treatment provides modest cardioprotection in the aged heart, reflected by improved post-ischemic electrical recovery without improvement of mechanical function. This effect appears to be partially mediated by reduced apoptosis, but independent of RISK pathway activation or autophagy. Aging thus alters the myocardial response to QCT, underscoring the importance of evaluating cardioprotective strategies in age-appropriate and clinically relevant experimental models.

### 5.2 Study limitations

Despite our study provides novel and important data regarding potential cardioprotective effects of QCT in aging heart, certain limitations should be taken into account when interpreting the data. First, only male animals were included in the study; therefore, the manuscript doesn´t address sex differences in the effects of QCT in aged heart. Sex differences encompass protective effects of the endocrine regulation in females; therefore, they may affect the cardiac effects of QCT treatment. Second, the Langendorff setup has a certain intrinsic limitation for evaluating the contractile function, as the ventricles are not filled within the retrograde perfusion mode. In this regard, the working heart model of Langendorff setup would better simulate real heart workload and function. Finally, only a single dose of QCT was used in the study; alternative doses or longer treatment might yield different outcomes. However, when standard body surface area–based conversion is applied, the QCT dose used in our study corresponds to human-equivalent doses that overlap with amounts commonly used in nutritional supplementation studies. Finally, since the aim of the study was to evaluate the effect of QCT in aged heart, the rats of only one age were used (20 months old at arrival). Using more age categories of animals might bring more detailed insight into the contribution of aging to the cardioprotection.

## Data Availability

The raw data supporting the conclusions of this article will be made available by the authors, without undue reservation.

## References

[1] North BJ Sinclair DA. The intersection between aging and cardiovascular disease. Circ Res. (2012) 110:1097–108. 10.1161/CIRCRESAHA.111.246876 22499900 PMC3366686

[2] Jia G Hill MA Sowers JR. Diabetic cardiomyopathy: an update of mechanisms contributing to this clinical entity. Circ Res. (2018) 122:624–38. 10.1161/CIRCRESAHA.117.311586 29449364 PMC5819359

[3] Goyal P Maurer MS Roh J. Aging in heart failure. JACC Hear Fail. (2024) 12:795–809. 10.1016/j.jchf.2024.02.021 PMC11331491 38597865

[4] Zuurbier CJ Bertrand L Beauloye CR Andreadou I Ruiz-Meana M Jespersen NR Cardiac metabolism as a driver and therapeutic target of myocardial infarction. J Cell Mol Med. (2020) 24:5937–54. 10.1111/jcmm.15180 32384583 PMC7294140

[5] Zhou H Toan S. Pathological roles of mitochondrial oxidative stress and mitochondrial dynamics in cardiac microvascular ischemia/reperfusion injury. Biomolecules. (2020) 10(1):85. 10.3390/biom10010085 31948043 PMC7023463

[6] Lacolley P Avril S Gáll T Balla G Balla J Michel J-B Aging in the vascular system: lessons from mechanobiology, computational approaches, and oxidative stress. Cardiovasc Res. (2025) 121:1566–81. 10.1093/cvr/cvaf137 40810190

[7] Bi F Gao C Guo H. Epigenetic regulation of cardiovascular diseases induced by behavioral and environmental risk factors: mechanistic, diagnostic, and therapeutic insights. FASEB bioAdvances. (2024) 6:477–502. 10.1096/fba.2024-00080 39512842 PMC11539034

[8] Abdellatif M Sedej S Carmona-Gutierrez D Madeo F Kroemer G. Autophagy in cardiovascular aging. Circ Res. (2018) 123:803–24. 10.1161/CIRCRESAHA.118.312208 30355077

[9] Berkers CR Maddocks ODK Cheung EC Mor I Vousden KH. Metabolic regulation by p53 family members. Cell Metab. (2013) 18:617–33. 10.1016/j.cmet.2013.06.019 23954639 PMC3824073

[10] Wang K Zhu Q Liu W Wang L Li X Zhao C Mitochondrial apoptosis in response to cardiac ischemia-reperfusion injury. J Transl Med. (2025) 23:125. 10.1186/s12967-025-06136-8 39875870 PMC11773821

[11] Shi H Gao Y Dong Z Yang J Gao R Li X GSDMD-Mediated Cardiomyocyte pyroptosis promotes myocardial I/R injury. Circ Res. (2021) 129:383–96. 10.1161/CIRCRESAHA.120.318629 34015941 PMC8291144

[12] Wang S-S Zhang X Ke Z-Z Zeng Y-X Wen X-Y Liu W-B Klotho attenuates D-galactose-induced cardiac aging through the ROS/NLRP3/pyroptosis pathway. J Mol Cell Cardiol. (2025) 208:35–48. 10.1016/j.yjmcc.2025.09.004 40930416

[13] Lacroix M Riscal R Arena G Linares LK Le Cam L. Metabolic functions of the tumor suppressor p53: implications in normal physiology, metabolic disorders, and cancer. Mol Metab. (2020) 33:2–22. 10.1016/j.molmet.2019.10.002 31685430 PMC7056927

[14] Zhu X-Z Qiu Z Lei S-Q Leng Y Li W-Y Xia Z-Y. The role of P53 in myocardial ischemia-reperfusion injury. Cardiovasc Drugs Ther. (2025) 39:195–209. 10.1007/s10557-023-07480-x 37389674

[15] Chen Q Thompson J Hu Y Das A Lesnefsky EJ. Cardiac specific knockout of p53 decreases ER stress-induced mitochondrial damage. Front Cardiovasc Med. (2019) 6:10. 10.3389/fcvm.2019.00010 30838215 PMC6389610

[16] Lee DJW Hodzic Kuerec A Maier AB. Targeting ageing with rapamycin and its derivatives in humans: a systematic review. lancet Heal Longev. (2024) 5:e152–62. 10.1016/S2666-7568(23)00258-1 38310895

[17] Eisenberg T Abdellatif M Schroeder S Primessnig U Stekovic S Pendl T Cardioprotection and lifespan extension by the natural polyamine spermidine. Nat Med. (2016) 22:1428–38. 10.1038/nm.4222 27841876 PMC5806691

[18] Börzsei D Sebestyén J Szabó R Lesi ZN Pálszabó A Pálszabó P Resveratrol as a promising polyphenol in age-associated cardiac alterations. Oxid Med Cell Longev. (2022) 2022:7911222. 10.1155/2022/7911222 35761875 PMC9233576

[19] Mury P Dagher O Fortier A Diaz A Lamarche Y Noly P-E Quercetin reduces vascular senescence and inflammation in symptomatic male but not female coronary artery disease patients. Aging Cell. (2025) 24:e70108. 10.1111/acel.70108 40375481 PMC12341813

[20] Zhang Y-M Zhang Z-Y Wang R-X. Protective mechanisms of quercetin against myocardial ischemia reperfusion injury. Front Physiol. (2020) 11:956. 10.3389/fphys.2020.00956 32848878 PMC7412593

[21] Barteková M Carnická S Pancza D Ondrejcáková M Breier A Ravingerová T. Acute treatment with polyphenol quercetin improves postischemic recovery of isolated perfused rat hearts after global ischemia. Can J Physiol Pharmacol. (2010) 88:465–71. 10.1139/y10-025 20555415

[22] Barteková M Šimončíková P Fogarassyová M Ivanová M Okruhlicová Ľ Tribulová N Quercetin improves postischemic recovery of heart function in doxorubicin-treated rats and prevents doxorubicin-induced matrix metalloproteinase-2 activation and apoptosis induction. Int J Mol Sci. (2015) 16:8168–85. 10.3390/ijms16048168 25872140 PMC4425074

[23] Chang X Zhang Q Huang Y Liu J Wang Y Guan X Quercetin inhibits necroptosis in cardiomyocytes after ischemia-reperfusion via DNA-PKcs-SIRT5-orchestrated mitochondrial quality control. Phytother Res. (2024) 38:2496–517. 10.1002/ptr.8177 38447978

[24] Albadrani GM BinMowyna MN Bin-Jumah MN El-Akabawy G Aldera H Al-Farga AM. Quercetin prevents myocardial infarction adverse remodeling in rats by attenuating TGF-*β*1/Smad3 signaling: different mechanisms of action. Saudi J Biol Sci. (2021) 28:2772–82. 10.1016/j.sjbs.2021.02.007 34012318 PMC8116976

[25] Farazi MM Rostamzadeh F Jafarinejad-Farsangi S Moazam Jazi M Jafari E Gharbi S. CircPAN3/miR-221/PTEN axis and apoptosis in myocardial infarction: quercetin’s regulatory effects. Gene. (2024) 909:148316. 10.1016/j.gene.2024.148316 38401834

[26] Shu Z Yang Y Yang L Jiang H Yu X Wang Y. Cardioprotective effects of dihydroquercetin against ischemia reperfusion injury by inhibiting oxidative stress and endoplasmic reticulum stress-induced apoptosis via the PI3K/akt pathway. Food Funct. (2019) 10:203–15. 10.1039/c8fo01256c 30525169

[27] Xiong D Wang X Wang H Chen X Li H Li Y Quercetin inhibits cardiomyocyte apoptosis via Sirt3/SOD2/mitochondrial reactive oxygen species during myocardial ischemia-reperfusion injury. Heliyon. (2024) 10:e39031. 10.1016/j.heliyon.2024.e39031 39568838 PMC11577236

[28] Shirakabe A Ikeda Y Sciarretta S Zablocki DK Sadoshima J. Aging and autophagy in the heart. Circ Res. (2016) 118:1563–76. 10.1161/CIRCRESAHA.116.307474 27174950 PMC4869999

[29] Hu S Lv L Hu W Shen J. Quercetin improves myocardial ischemia-reperfusion injury by regulating macrophage M2 polarization through bcl-2/beclin-1 complex. Eur J Med Res. (2025) 30:780. 10.1186/s40001-025-03077-2 40836312 PMC12369085

[30] Mrkus L Batinić J Bjeliš N Jakas A. Synthesis and biological evaluation of quercetin and resveratrol peptidyl derivatives as potential anticancer and antioxidant agents. Amino Acids. (2019) 51:319–29. 10.1007/s00726-018-2668-6 30392096

[31] Ferenczyova K Kalocayova B Kindernay L Jelemensky M Balis P Berenyiova A Quercetin exerts age-dependent beneficial effects on blood pressure and vascular function, but is inefficient in preventing myocardial ischemia-reperfusion injury in zucker diabetic fatty rats. Molecules. (2020) 25:187. 10.3390/molecules25010187 31906454 PMC6983107

[32] Bartekova M Radosinska J Pancza D Barancik M Ravingerova T. Cardioprotective effects of quercetin against ischemia-reperfusion injury are age-dependent. Physiol Res. (2016) 65:S101–7. 10.33549/physiolres.933390 27643931

[33] Kmecova J Klimas J. Heart rate correction of the QT duration in rats. Eur J Pharmacol. (2010) 641:187–92. 10.1016/j.ejphar.2010.05.038 20553920

[34] Bazett HC. The time relations of the blood-pressure changes after excision of the adrenal glands, with some observations on blood volume changes. J Physiol. (1920) 53:320–39. 10.1113/jphysiol.1920.sp001881 16993419 PMC1405575

[35] Bartosova L Horvath C Galis P Ferenczyova K Kalocayova B Szobi A Quercetin alleviates diastolic dysfunction and suppresses adverse pro-hypertrophic signaling in diabetic rats. Front Endocrinol. (2022) 13:1029750. 10.3389/fendo.2022.1029750 PMC9772025 36568083

[36] Sengupta P. The laboratory rat: relating its age with human’s. Int J Prev Med. (2013) 4:624–30. 23930179 PMC3733029

[37] Puymirat E Simon T Cayla G Cottin Y Elbaz M Coste P Acute myocardial infarction: changes in patient characteristics, management, and 6-month outcomes over a period of 20 years in the FAST-MI program (French registry of acute ST-elevation or non-ST-elevation myocardial infarction) 1995 to 2015. Circulation. (2017) 136:1908–19. 10.1161/CIRCULATIONAHA.117.030798 28844989

[38] Liu C Huang J Qiu J Jiang H Liang S Su Y Quercitrin improves cardiac remodeling following myocardial infarction by regulating macrophage polarization and metabolic reprogramming. Phytomedicine. (2024) 127:155467. 10.1016/j.phymed.2024.155467 38447360

[39] Annapurna A Reddy CS Akondi RB Rao SRC. Cardioprotective actions of two bioflavonoids, quercetin and rutin, in experimental myocardial infarction in both normal and streptozotocin-induced type I diabetic rats. J Pharm Pharmacol. (2009) 61:1365–74. 10.1211/jpp/61.10.0014 19814870

[40] Yellon DM Beikoghli Kalkhoran S Davidson SM. The RISK pathway leading to mitochondria and cardioprotection: how everything started. Basic Res Cardiol. (2023) 118:22. 10.1007/s00395-023-00992-5 37233787 PMC10220132

[41] Franco-Vadillo A Toledo-Blass M Rivera-Herrera Z Guevara-Balcazar G Orihuela-Rodriguez O Morales-Carmona JA Cannabidiol-mediated RISK PI3K/AKT and MAPK/ERK pathways decreasing reperfusion myocardial damage. Pharmacol Res Perspect. (2021) 9:e00784. 10.1002/prp2.784 34176244 PMC8236079

[42] Yang S Li H Tang L Ge G Ma J Qiao Z Apelin-13 protects the heart against ischemia-reperfusion injury through the RISK-GSK-3β-mPTP pathway. Arch Med Sci. (2015) 11:1065–73. 10.5114/aoms.2015.54863 26528352 PMC4624751

[43] Kim SJ Li M Jeong CW Bae HB Kwak SH Lee SH Epigallocatechin-3-gallate, a green tea catechin, protects the heart against regional ischemia-reperfusion injuries through activation of RISK survival pathways in rats. Arch Pharm Res. (2014) 37:1079–85. 10.1007/s12272-013-0309-x 24307060

[44] Ferenczyová K Kindernay L Vlkovičová J Kaločayová B Rajtík T Barteková M. Pharmacology of catechins in ischemia-reperfusion injury of the heart. Antioxidants. (2021) 10(9):1390. 10.3390/antiox10091390 34573022 PMC8465198

[45] Jeong C-W Yoo KY Lee SH Jeong HJ Lee CS Kim SJ. Curcumin protects against regional myocardial ischemia/reperfusion injury through activation of RISK/GSK-3β and inhibition of p38 MAPK and JNK. J Cardiovasc Pharmacol Ther. (2012) 17:387–94. 10.1177/1074248412438102 22396328

[46] Shanmugam K Boovarahan SR Prem P Sivakumar B Kurian GA. Fisetin attenuates myocardial ischemia-reperfusion injury by activating the reperfusion injury salvage kinase (RISK) signaling pathway. Front Pharmacol. (2021) 12:566470. 10.3389/fphar.2021.566470 33762932 PMC7982788

[47] Liu H Guo X Chu Y Lu S. Heart protective effects and mechanism of quercetin preconditioning on anti-myocardial ischemia reperfusion (IR) injuries in rats. Gene. (2014) 545:149–55. 10.1016/j.gene.2014.04.043 24769323

[48] Park E-S Kang JC Jang YC Park JS Jang SY Kim D-E Cardioprotective effects of rhamnetin in H9c2 cardiomyoblast cells under H₂O₂-induced apoptosis. J Ethnopharmacol. (2014) 153:552–60. 10.1016/j.jep.2014.02.019 24607510

[49] Roslan J Giribabu N Karim K Salleh N. Quercetin ameliorates oxidative stress, inflammation and apoptosis in the heart of streptozotocin-nicotinamide-induced adult male diabetic rats. Biomed Pharmacother. (2017) 86:570–82. 10.1016/j.biopha.2016.12.044 28027533

[50] Chen Y-F Qiu Q Wang L Li X-R Zhou S Wang H Quercetin ameliorates myocardial injury in diabetic rats by regulating autophagy and apoptosis through AMPK/mTOR signaling pathway. Am J Chin Med. (2024) 52:841–64. 10.1142/S0192415X24500344 38716618

[51] Li C Wang T Zhang C Xuan J Su C Wang Y. Quercetin attenuates cardiomyocyte apoptosis via inhibition of JNK and p38 mitogen-activated protein kinase signaling pathways. Gene. (2016) 577:275–80. 10.1016/j.gene.2015.12.012 26680104

[52] Yan M Lin K Huang D Li J Qu X Chen K. Semaglutide attenuates pathological electrophysiological remodeling in diabetic cardiomyopathy via restoring Cx43 expression. Endocrine. (2024) 84:969–79. 10.1007/s12020-024-03823-2 38647981

[53] Liang Y Zheng B Li J Shi J Chu L Han X Crocin ameliorates arsenic trioxide-induced cardiotoxicity via Keap1-Nrf2/HO-1 pathway: reducing oxidative stress, inflammation, and apoptosis. Biomed Pharmacother. (2020) 131:110713. 10.1016/j.biopha.2020.110713 32920515

[54] Wang S Ji F Gao X Li Z Lv S Zhang J Tyrosine kinase inhibitor lenvatinib causes cardiotoxicity by inducing endoplasmic Reticulum stress and apoptosis through activating ATF6, IRE1*α* and PERK signaling pathways. Recent Pat Anticancer Drug Discov. (2025) 20:168–84. 10.2174/0115748928265981231204044653 38994620

[55] Shang X Ji X Dang J Wang L Sun C Liu K *α*-asarone induces cardiac defects and QT prolongation through mitochondrial apoptosis pathway in zebrafish. Toxicol Lett. (2020) 324:1–11. 10.1016/j.toxlet.2020.02.003 32035120

[56] Taneike M Yamaguchi O Nakai A Hikoso S Takeda T Mizote I Inhibition of autophagy in the heart induces age-related cardiomyopathy. Autophagy. (2010) 6:600–6. 10.4161/auto.6.5.11947 20431347

[57] Granado M Amor S Martín-Carro B Guerra-Menéndez L Tejera-Muñoz A González-Hedström D García-Villalón ÁL. Caloric restriction attenuates aging-induced cardiac insulin resistance in male wistar rats through activation of PI3K/akt pathway. Nutr Metab Cardiovasc Dis. (2019) 29:97–105. 10.1016/j.numecd.2018.09.005 30497927

