# Supplementary material for: Chronic quercetin supplementation modulates cardiac function and signaling pathways in aged male Wistar rat hearts subjected to ischemia-reperfusion
Source: Front Cardiovasc Med. 2026 Apr 28;13:1745113. doi: 10.3389/fcvm.2026.1745113 (PMC13160773; doi:10.3389/fcvm.2026.1745113)
Supplement: Supplementary file 1 [file Table1.docx]

Chronic Quercetin Supplementation Modulates Cardiac Function and Signaling Pathways in Aged Male Wistar Rat Hearts Subjected to Ischemia-Reperfusion

Jakub Strapec^1^, Lucia Kindernay^1^, Eva Kralova^2^, Katarina Krsteva^1^, Peter Pokreisz^3^, Monika Bartekova^1,4,*^, Tomas Rajtik^1,2^, Kristina Ferenczyova^1,2^

^1^Institute for Heart Research, Centre of Experimental Medicine, Slovak Academy of Sciences, Bratislava, Slovakia

^2^Department of Pharmacology and Toxicology, Faculty of Pharmacy, Comenius University, Bratislava, Slovakia

^3^Center for Biomedical Research and Translational Surgery, Medical University of Vienna, Vienna, Austria

^4^Institute of Physiology, Faculty of Medicine, Comenius University, Bratislava, Slovakia

***Correspondence:**Dr. Monika Bartekova, PhD.
monika.bartekova@savba.sk

Keywords: ischemia-reperfusion, aging, quercetin, apoptosis, autophagy

Known cardiovascular effects of quercetin

| Study type | Effect | Experimental model | Reference |
| --- | --- | --- | --- |
| In vitro | Antioxidant effects | Cardiomyocytes | [20] |
|  | Anti-apoptotic effects | H9c2 cardiomyocytes | [23,27] |
|  | RISK pathway activation | Cardiomyocytes | [26] |
|  | Inhibition of necroptosis | Cardiomyocytes | [23] |
| In vivo | Cardioprotection against ischemia–reperfusion | Isolated perfused hearts | [21, 22] |
|  | Reduced infarct size | Rat myocardial infarction models | [24, 25] |
|  | Promotion of M2 macrophage polarization | Myocardial ischemia–reperfusion model | [29] |

**Supplamentary Table 1. Known cardiovascular effects of quercetin.** QCT, quercetin; I/R, ischemia–reperfusion; MIRI, myocardial ischemia–reperfusion injury. Summarizes reported in vitro and in vivo cardiovascular effects of quercetin in experimental models. In vitro effects were identified in isolated cardiomyocytes or endothelial cells, whereas in vivo effects were demonstrated in experimental models of myocardial ischemia–reperfusion or myocardial infarction.
