# Supplementary figures and images for: Chronic quercetin supplementation modulates cardiac function and signaling pathways in aged male Wistar rat hearts subjected to ischemia-reperfusion
Source: Front Cardiovasc Med. 2026 Apr 28;13:1745113. doi: 10.3389/fcvm.2026.1745113 (PMC13160773; doi:10.3389/fcvm.2026.1745113)

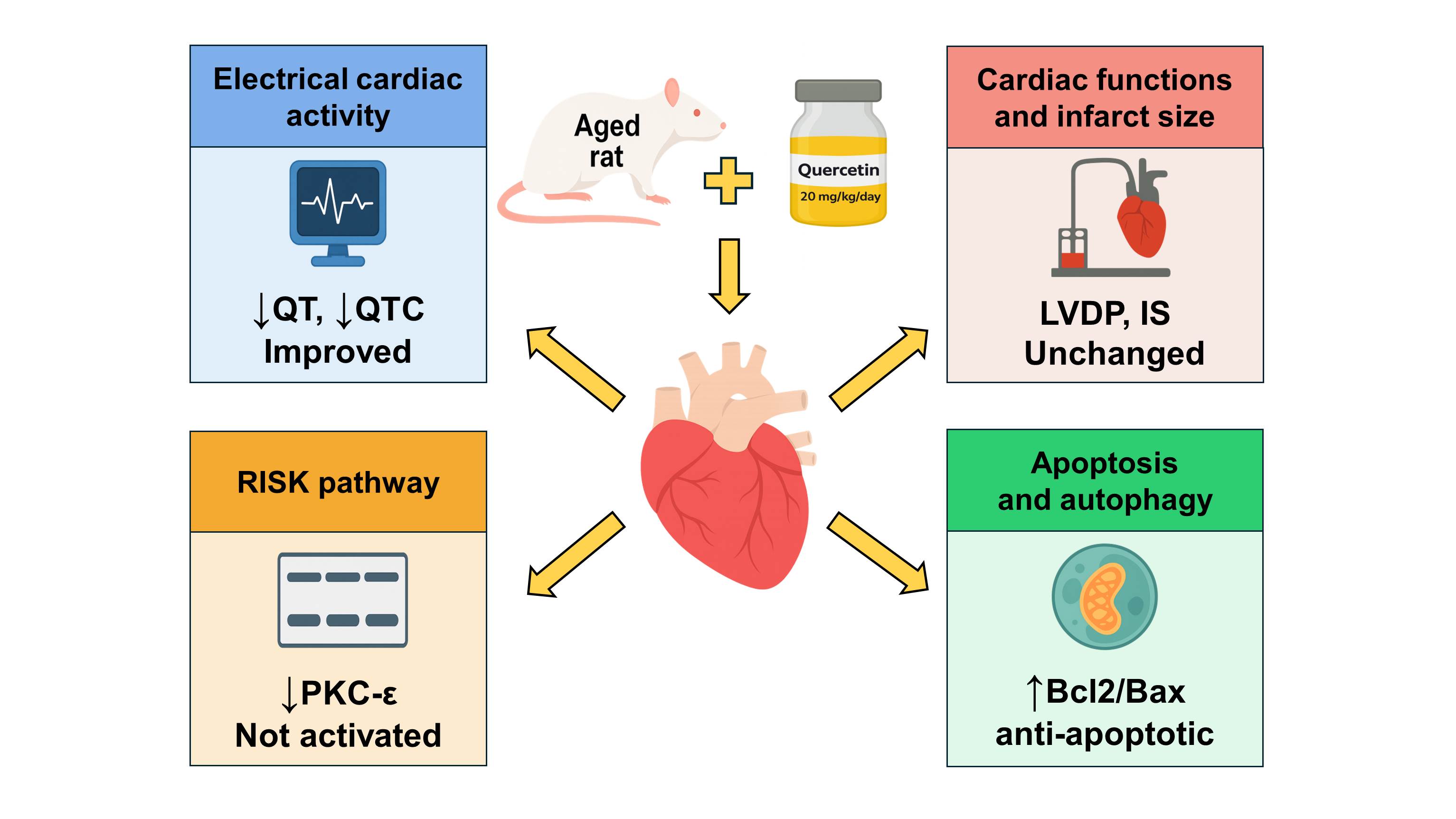

Supplement: Supplementary file 2 [file Image1.jpeg]
